# An urban patrol case urgency classification method based on self-supervised clustering of a graph neural network

**DOI:** 10.1371/journal.pone.0360432

**Published:** 2026-10-09

**Authors:** Yirui Jiang, Hao Wu, Tiejun Yang, Jiayu Sun, Hongwei Li, Wenjie Zhu

**Affiliations:** 1 School of Artificial Intelligence and Big Data, Henan University of Technology, Zhengzhou, Henan, China; 2 School of Computer and Artificial Intelligence, Zhengzhou University, Zhengzhou, China; National University of Defense Technology, CHINA

## Abstract

Accurate assessment of urban patrol cases plays an important role in maintaining urban safety and improving the efficiency of urban governance. The hazard level of urban patrol cases can be reflected by the urgency level and prioritizing high-urgency cases can help reduce potential risks to public safety. However, it remains challenging to assess the urgency level of urban patrol cases when reliable urgency labels are limited or unavailable. To address this problem, this paper proposes an urban patrol case urgency classification method based on self-supervised clustering of a graph neural network. First, a case-relationship graph is constructed from urban patrol cases. A graph attention-based autoencoder is then pretrained to learn node representations, and K-means is used to initialize cluster centers. Finally, representation learning and clustering are jointly optimized to identify urgency-oriented clusters without using manual labels during training. Experimental validation was conducted using 6126 urban patrol case records from Zhengzhou, China. The proposed method achieved a Recall of 0.7458, Precision of 0.7465, ACC of 0.7424, F1 of 0.7403, and NMI of 0.4517. Compared with TADW, the strongest baseline, the proposed method improved these metrics by 0.0617, 0.0479, 0.0650, 0.0690, and 0.0407, respectively. These results demonstrate that the proposed method can effectively learn urgency-related latent representations and improve the classification quality of urban patrol cases.

## Introduction

As urban processes grow, they pose challenges to urban safety. Urban management personnel identified issues in urban construction through street patrols [1–3]. Patrol case data are formed by a number of patrol cases distributed in a continuous data space of high-event-density regions [4–6]. Multiple urban patrol cases may occur in the same period and can be prioritized according to the urgency level of the event to address events with high levels of harm, which can reduce the impact on human safety.

A graph is a data structure used to model objects and their relationships [7]. With the development of artificial intelligence, graph-based methods have become important tools in many fields, including social sciences, biotechnology, knowledge graphs, and other research areas [8–11]. Graph analytics mainly focuses on tasks such as node classification, link prediction, and clustering [12,13]. As an effective deep learning approach for graph-structured data, graph neural networks (GNNs) have been widely used because of their strong ability to capture both node attributes and structural relationships.

Urban patrol case data can be naturally represented as attributed graphs, where each case is treated as a node and relationships between cases are represented as edges [14,15]. Node attributes describe the individual characteristics of patrol cases, while graph edges capture pairwise relationships among cases. Through iterative neighborhood aggregation, GNNs transform nodes into embeddings that integrate both their own attributes and information from neighboring nodes. To reduce computational cost, practical GNN implementations often limit the number of aggregation iterations or use sampling strategies.

Clustering divides data objects into groups with similar characteristics [16] and can be regarded as an unsupervised classification strategy [17]. It has been widely used in urban patrol and public safety analysis. Hajela et al. [18] proposed a clustering-based hotspot prediction method using patrol case data from San Francisco, USA. Sivaranjani et al. [19] applied K-nearest neighbor, K-means clustering, hierarchical clustering, and DBSCAN to predict patrol-related incidents in six cities in Tamil Nadu, India.

Graph clustering further extends traditional clustering by grouping nodes in a graph into disjoint clusters. It has been widely applied to community detection, group segmentation, and functional group discovery [15,16,20–23]. For attributed graph clustering, an important issue is how to jointly capture graph structural relationships and node content information. Earlier studies mainly used shallow graph clustering methods. Girvan et al. [24] detected communities using centrality indices, while Hastings et al. [20] applied information propagation for community detection. In addition, embedding-based methods, relational topic models, and co-clustering methods have been used to process structural and content information [25–27].

With the development of deep learning, GNN-based graph clustering has achieved significant progress. Wu et al. [28] proposed a GNN-based topic clustering framework, Cheng et al. [29] developed a multi-view attribute graph convolutional network for clustering, and Shi et al. [30] introduced a graph attention network-based clustering architecture to reduce redundant connections through progressive clustering. However, these methods are mainly designed for general graph clustering tasks, and their application to urban patrol case urgency classification remains limited.

A GNN processes graph-structured data composed of nodes and edges [15] and can handle complex structured data such as social networks, chemical compounds, and protein structures [31]. In a typical GNN, each node is associated with an initial feature vector, and its representation is updated by aggregating information from neighboring nodes. The updated node embeddings can then be used for downstream tasks [14,32,33]. As an important variant of GNNs, the graph attention network (GAT) introduces an attention mechanism to assign different weights to neighboring nodes [34]. By calculating attention coefficients between a node and its neighbors, GAT can adaptively measure the importance of different neighboring nodes and obtain more informative node representations [35–40].Urban event datasets can be clustered through a vector embedding process that involves each data point, where each case in the dataset is transformed into a vector in a high-dimensional space. In this dataset, temporal and coordinate information can be used to embed each case in space-time. This means that events that are close to each other in time and space will be embedded close to each other in a high-dimensional vector space. By using vector embedding to cluster the dataset, city managers can observe patterns and trends in the development of patrol cases and propose more targeted and effective response strategies.

Although GNN-based clustering has been widely studied, most existing methods assume that the graph structure is already available and focus on general node-clustering tasks. In contrast, urban patrol data are originally stored as heterogeneous tabular records. The novelty of this study lies in constructing an attributed case-relationship graph directly from patrol records and formulating urgency classification as an unsupervised graph-clustering problem. The proposed framework further integrates graph attention-based autoencoder pretraining, K-means initialization, and joint representation–clustering optimization, while historical urgency labels are used only for post-hoc evaluation. In this work, an unsupervised clustering strategy is used for urban patrol case urgency classification. An attributed case-relationship graph is first constructed from patrol records using event attributes, spatial proximity, and event-type similarity. A graph attention-based autoencoder is then employed to jointly learn node-content and graph-structure information. Finally, a self-supervised clustering module is introduced to jointly refine the latent representations and clustering assignments.

The contributions of this study are as follows:

- An urgency assessment method for urban patrol cases is proposed, in which the urgency of events is assessed via a clustering method with unsupervised learning.
- An attributed case-relationship graph is constructed from raw urban patrol data to jointly represent case attributes and contextual dependencies among events.
- A self-supervised deep graph clustering framework is developed, in which a graph attention-based autoencoder is first pretrained to learn structural and attribute-aware representations, and K-means is subsequently used to initialize the cluster centers.
- An end-to-end joint optimization strategy is introduced to iteratively refine node representations and clustering assignments.
- The proposed method achieves a Recall of 0.7458, Precision of 0.7465, ACC of 0.7424, and F1-score of 0.7403, confirming its effectiveness for urban patrol case urgency classification.

## Materials and methods

### Study region

Zhengzhou, China, was selected as the study region in this work. Urban management in Zhengzhou involves diverse types of patrol cases occurring across different locations and time periods, including construction management, cityscape management, emergencies, publicity and advertisement, and other urban governance issues. The spatial distribution, event category, occurrence time, and other contextual characteristics of these cases provide useful information for investigating potential urgency-related patterns.

The urban patrol cases in the study region contain both spatial and non-spatial attributes. Spatial attributes, such as longitude and latitude, describe the geographic location of individual cases, whereas non-spatial attributes, including event category and case description, characterize their semantic properties. In addition, occurrence time provides temporal information for describing the contextual characteristics of patrol events. These heterogeneous attributes make Zhengzhou a suitable real-world setting for constructing an attributed case-relationship graph and evaluating the proposed self-supervised deep graph clustering framework.

In this study, urban patrol case records collected in Zhengzhou were used for model construction and experimental analysis. Spatial relationships among patrol cases were determined directly from the geographic coordinates of individual events, while semantic relationships were established according to event-type information. Therefore, the proposed case-relationship graph was constructed directly from the patrol case records and did not rely on road-network topology or external map information. Using this real-world urban patrol dataset, this study evaluates whether the proposed framework can learn urgency-related latent representations and identify clustering structures that correspond to the recorded urgency categories.

### Datasets

Urban patrol case records collected in Zhengzhou, China, were used as the primary dataset for model construction and experimental analysis. The records contain non-spatial attributes, such as case codes, event categories, and event descriptions, as well as spatial attributes, including geographic coordinates, and temporal attributes, including occurrence times. From October to December 2018, a total of 6,126 urban patrol cases were collected, covering construction management, cityscape management, emergencies, publicity and advertisement, and other urban governance issues. Basic road network data from 2018 were additionally used only for geographic visualization of the study region and were not used as model inputs or for case-relationship graph construction.

To provide a more comprehensive description of the dataset used for urban patrol case urgency classification, several supplementary tables are presented. Table 1 presents representative urban patrol case records used for urgency classification, including case number, event category, occurrence time, longitude, latitude, urgency label, and event details. For privacy reasons, specific latitude and longitude coordinates and times are not provided. Latitude and longitude are displayed to only the last two digits, and times are displayed only to the hour.

**Table 1:** Examples of urban patrol case records.

Case No.	Case Category	Time	Longitude	Latitude	Urgency label	Details
809848	Fires and other emergencies	2018/10/14 17:02:32	113.53	34.80	5	Building fire
269598	Water supply and drainage	2018/11/27 15:48:54	113.60	34.73	4	Pipe burst
380844	Road-related	2018/11/17 11:04:06	113.62	34.74	3	Road flooding
250187	Road-related	2018/12/7 7:52:12	113.59	34.73	2	Road debris
70304	Illegal ads and flyers	2018/12/18 11:15:17	113.61	34.70	1	Illegal Ad.

Fig 1 illustrates the distribution of urban patrol cases across different case categories. Road-related cases account for the largest proportion of the dataset, with 1,184 cases (19.3%), followed by other cases with 1,162 cases (19.0%) and trash-related cases with 793 cases (12.9%). In contrast, fires and other emergencies account for only 7 cases (0.1%).

**Fig 1:**
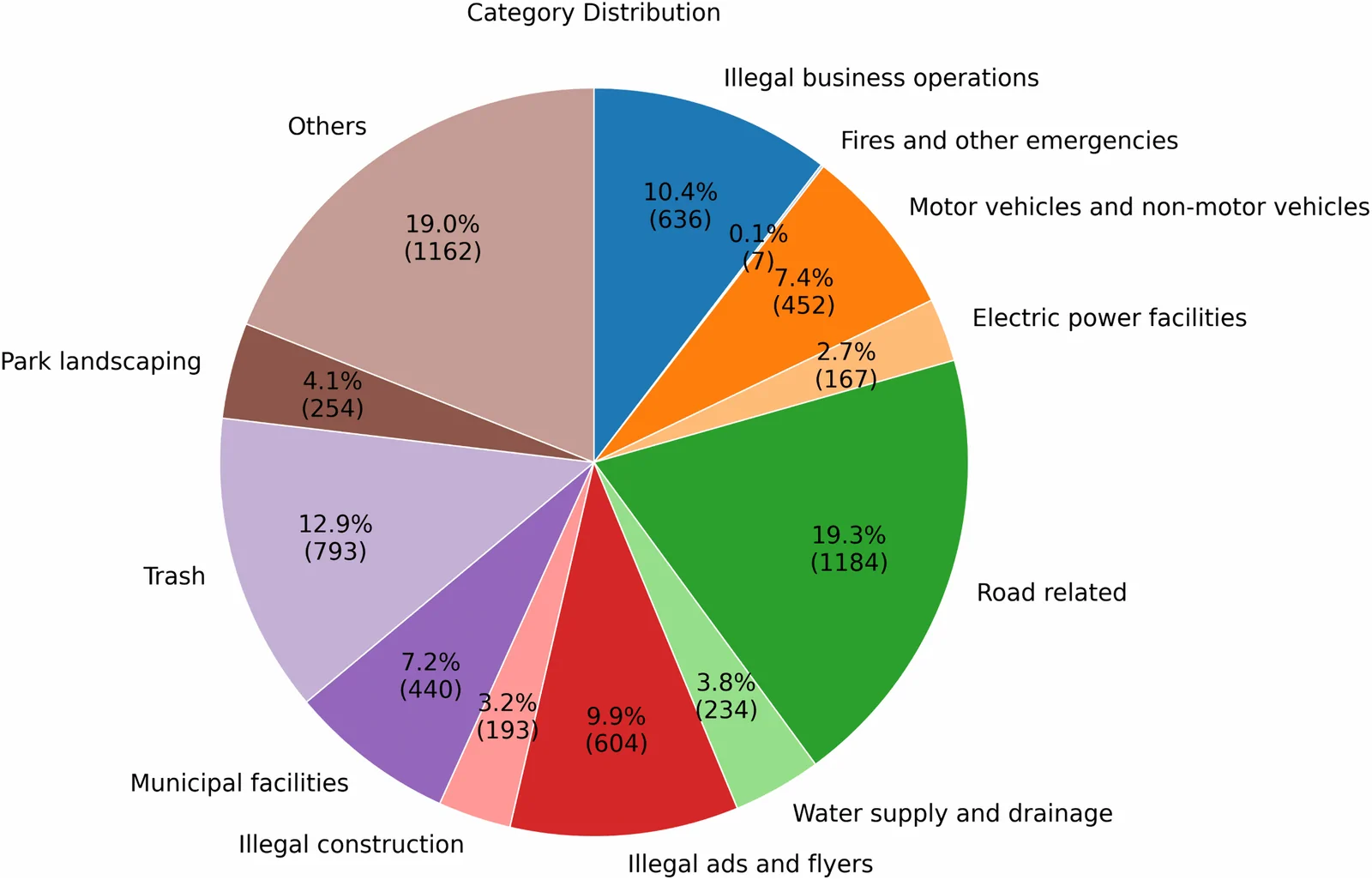
Distribution of urban patrol cases across different event categories.

As shown in Fig 2, the urgency labels in the original records were divided into five levels according to the response-priority scheme used in urban patrol management. Level 1 represents the lowest response priority, whereas Level 5 represents the highest response priority, with Levels 2–4 indicating progressively increasing levels of urgency. These categories were predefined in the historical administrative records and were not newly assigned or modified in this study. The recorded urgency levels were excluded from model training and were used only as reference categories for post-hoc external evaluation.

**Fig 2:**
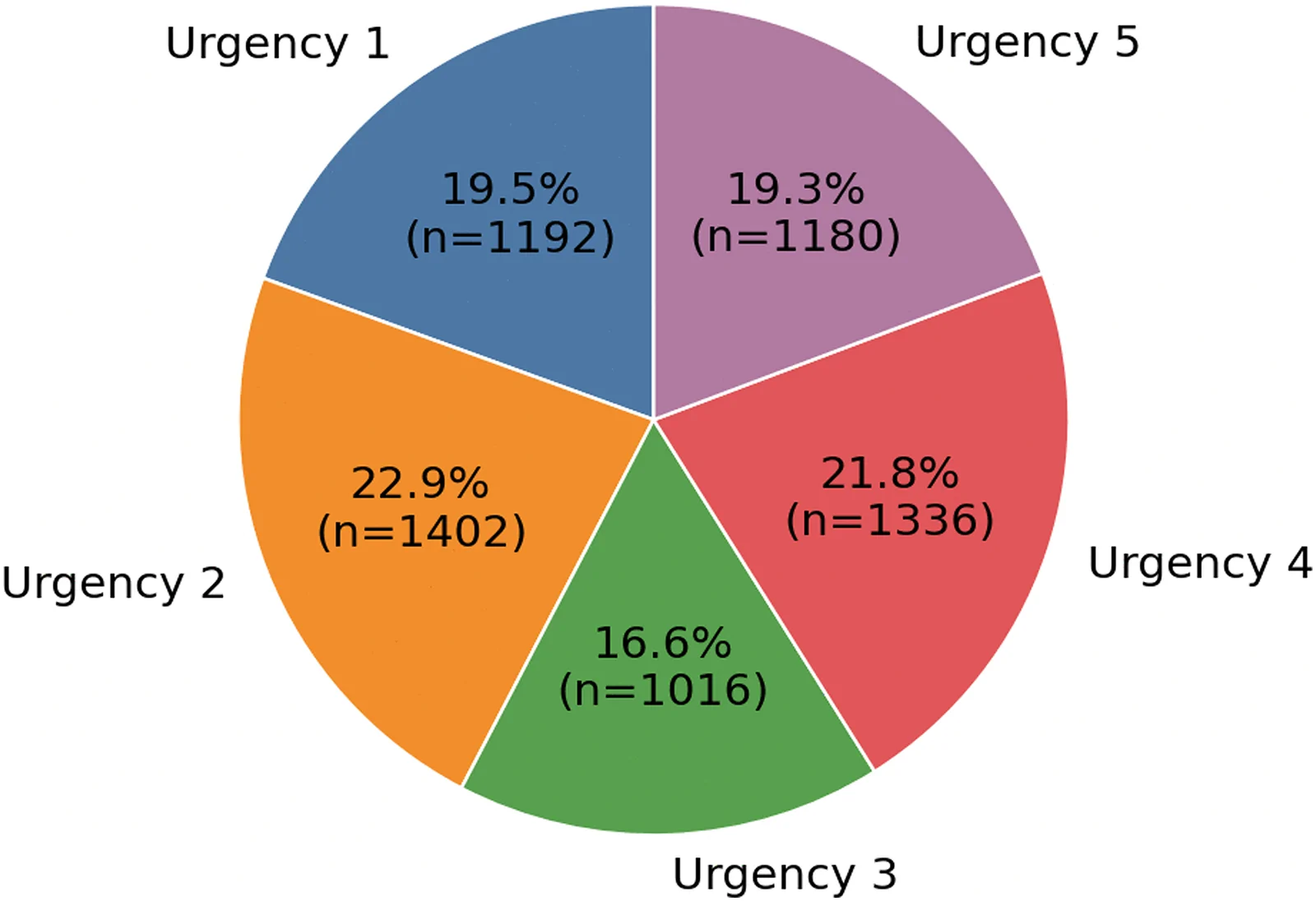
Distribution of urban patrol cases across different urgency levels.

### Methods

#### Overview of the proposed framework.

In this study, we propose a self-supervised deep graph clustering framework for urban patrol case urgency classification, as illustrated in Fig 3. The proposed framework consists of three main stages: case-relationship graph construction, graph attention-based autoencoder pretraining, and self-supervised clustering with joint optimization. Unlike conventional supervised classification methods, historical urgency labels are not used during representation learning or clustering optimization, but are retained only for post-hoc external evaluation.

**Fig 3:**
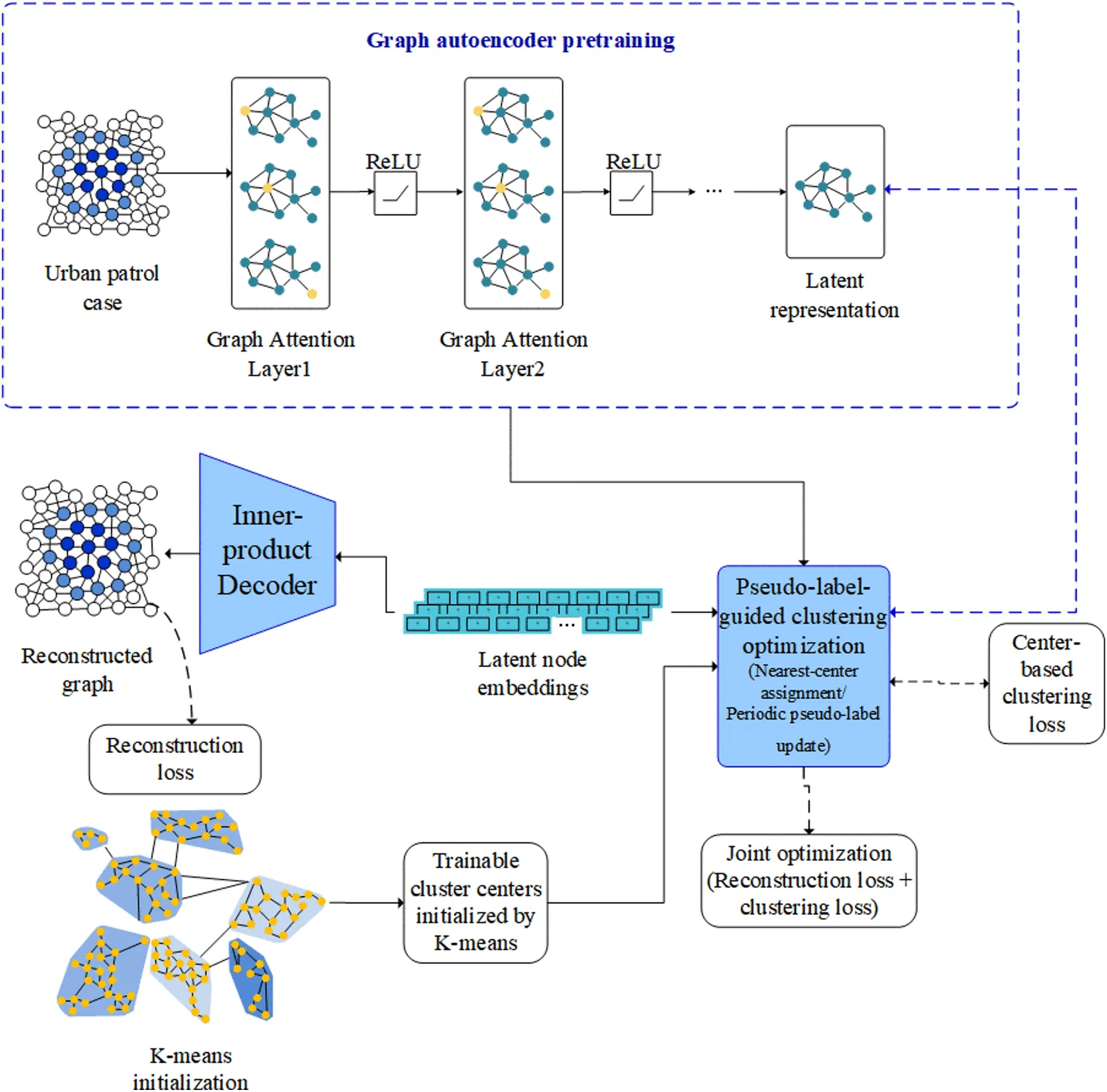
Framework of the proposed self-supervised deep graph clustering method.

First, the preprocessed urban patrol records are transformed into an attributed case-relationship graph. Each patrol case is represented as a node with attributes describing its event category, occurrence time, geographic location, administrative-region information, and other available pre-decision contextual information. The relationships among cases are established according to spatial proximity and event-type similarity. Therefore, the event graph is explicitly constructed from the original patrol records before graph neural network learning, rather than being generated by the GNN itself.

Second, a graph attention-based autoencoder is pretrained on the constructed case graph to learn latent node representations. The GAT encoder integrates node attributes with information from spatially and semantically related neighboring cases through attention-weighted aggregation, while an inner-product decoder reconstructs the graph structure from the learned embeddings. This unsupervised pretraining stage provides an informative and stable latent representation for subsequent clustering optimization without using historical urgency labels.

Third, K-means is applied to the pretrained node embeddings to initialize the cluster centers and cluster assignments. The initialized cluster assignments are treated as pseudo-labels rather than historical urgency labels. During the subsequent joint optimization stage, the graph encoder, decoder, and cluster centers are optimized simultaneously using a combination of graph reconstruction loss and clustering loss. The pseudo-labels are periodically updated according to the refined latent representations, allowing the learned embedding space and clustering structure to be progressively optimized toward more compact and separable urgency-related groups.

After convergence, the final cluster assignments are obtained according to the optimized cluster centers. Historical urgency labels are introduced only at this stage to establish the correspondence between discovered clusters and recorded urgency categories through Hungarian matching and to calculate external clustering evaluation metrics. Therefore, the entire representation-learning and clustering process remains unsupervised with respect to the recorded urgency labels.

The decision to formulate urgency classification as an unsupervised clustering task rather than a conventional supervised classification problem is motivated by both practical and conceptual considerations. In real urban-management scenarios, large-scale expert-labeled urgency data is rarely available, and the labels themselves are inherently subjective — different dispatchers frequently assign different urgency levels to similar cases, leading to inconsistent and noisy supervision signals. Beyond this practical limitation, urgency levels can be regarded as a latent grouping structure embedded in the case-feature distribution: patrol cases that share similar spatiotemporal characteristics, event types, and contextual attributes tend to require similar response priorities. Clustering therefore provides a principled means of recovering this latent structure, a perspective consistent with established practice in adjacent domains where severity, priority, or risk levels are routinely inferred through unsupervised grouping. To further ensure that the discovered clusters are semantically meaningful rather than arbitrary partitions, the proposed framework couples clustering with a graph attention–based autoencoder in an end-to-end fashion.

#### Data preprocessing and feature construction.

Before constructing the case-relationship graph, the raw urban patrol case records were preprocessed and transformed into structured model inputs. The preprocessing procedure included duplicate removal, abnormal-value correction, field-format standardization, and missing-value handling. Records with severe missing information in the attributes required for graph construction were excluded, whereas minor missing values were processed according to the attribute type and data distribution. Geographic coordinates outside the study area and records with invalid occurrence times were regarded as abnormal records and were corrected or removed after verification.

To prevent target leakage, only attributes available before the urgency level was assigned were retained as model inputs. These attributes included the reported event category, occurrence time, geographic location, administrative-region information, and other available pre-decision contextual attributes. The recorded urgency label was excluded from the node-feature matrix. Variables produced during or after urgency classification, including response priority, assigned urgency level, disposal outcome, processing status, response time, and completion information, were not used in feature construction, graph construction, representation learning, or clustering optimization.

Categorical attributes, such as event category and administrative region, were converted into numerical representations. Temporal features were extracted from the occurrence time to characterize the occurrence pattern of each event. Specifically, the month, day, weekday, and hour information available in the original records were transformed into numerical temporal attributes. Spatial features were derived from the longitude and latitude of each event and were used to describe its geographic location within the study region. Continuous features were normalized to reduce the influence of differences in measurement scales.

For an urban patrol event $v_{i}$, its initial node-feature vector is defined as


$$\begin{array}{c} x_{i}=\left[x_{i}^{\text{type}},x_{i}^{\text{time}},x_{i}^{\text{spatial}},x_{i}^{\text{region}},x_{i}^{\text{context}}\right] \end{array}$$ (1)

where $x_{i}^{\text{type}}$ represents the encoded event-category features, $x_{i}^{\text{time}}$ represents the temporal features extracted from the occurrence time, $x_{i}^{\text{spatial}}$ represents the normalized geographic features, $x_{i}^{\text{region}}$ represents the encoded administrative-, e.g.,ion information, and $x_{i}^{\text{context}}$ represents other contextual attributes available before urgency classification. If no additional contextual attributes were used, $x_{i}^{\text{context}}$ was omitted from the final feature vector.

After encoding and normalization, the feature vectors of all events were organized into the node-feature matrix


$$\begin{array}{c} X={\left[x_{\text{1}},x_{\text{2}},\ldots ,x_{N}\right]}^{\text{T}}\in R^{N\times D} \end{array}$$ (2)

where N denotes the number of urban patrol cases and D denotes the final node-feature dimension. The resulting node-feature matrix was subsequently combined with the case relationships defined by spatial proximity and event-type similarity to construct the attributed case-relationship graph. Historical urgency labels were retained separately and were used only after model convergence for cluster-to-category matching and external clustering evaluation.

#### Construction of the case-relationship graph.

In this section, the original urban patrol records are transformed into a case relationship graph. Under the transductive clustering setting, all observed patrol cases are used to construct a single graph. Each urban patrol case is regarded as a node, and the relationships among different nodes are established according to spatial proximity and event-type similarity. The overall construction procedure is illustrated in Fig 4, including the transformation from the original patrol records to spatial and semantic relationships and their integration into the final attributed case-relationship graph.

**Fig 4:**
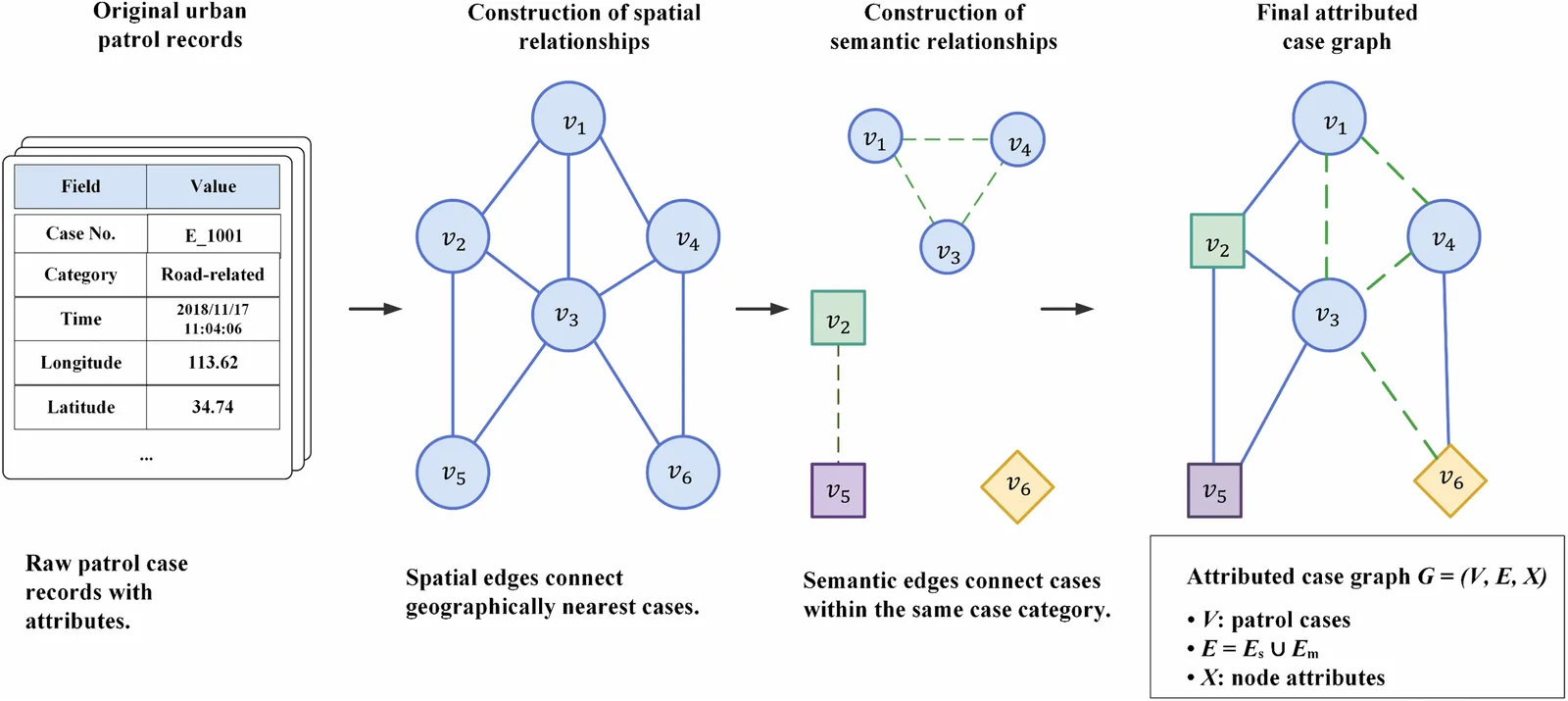
Construction of the attributed case-relationship graph.

The case relationship graph is defined as


$$\begin{array}{c} G=\left(V,E,X\right) \end{array}$$ (3)

where V denotes the set of event nodes, E represents the set of edges, and X denotes the node feature matrix. Each node corresponds to an individual urban patrol event. Its initial feature vector consists of the encoded event category, occurrence time, geographic coordinates, and other available contextual attributes:


$$\begin{array}{c} x_{i}=\left[x_{i}^{\text{type}},x_{i}^{\text{time}},x_{i}^{\text{lon}},x_{i}^{\text{lat}},x_{i}^{\text{context}}\right] \end{array}$$ (4)

The recorded urgency labels are not included in the node feature matrix and are not used during graph construction.

The case relationship graph is constructed based on both spatial proximity and case-type similarity. Spatial relationships are introduced because urban patrol cases occurring in nearby locations may share similar environmental conditions, infrastructure characteristics, administrative contexts, and response-resource constraints. The geographic distance between two event nodes $v_{i}$ and $v_{j}$ is calculated as


$$\begin{array}{c} d_{ij}=\text{Distance}\left[\left({\text{lon}}_{i},{\text{lat}}_{i}\right),\left({\text{lon}}_{j},{\text{lat}}_{j}\right)\right] \end{array}$$ (5)

where $d_{ij}$ denotes the geographic distance between the two cases. For each node, a predefined number of geographically nearest nodes are selected to establish spatial connections. The spatial adjacency matrix is defined as


$$\begin{array}{c} A_{ij}^{\text{spa}}=\left\{ \begin{array}{cc} \text{1}, & v_{j}\in N_{i}^{\text{spa}}\text{ or }v_{i}\in N_{j}^{\text{spa}},\\ \text{0}, & \text{otherwise}, \end{array}\right. \end{array}$$ (6)

where $N_{i}^{\text{spa}}$ denotes the spatial-neighbor set of node $v_{i}$. The spatial relationships are symmetrized to form undirected edges.

In addition to spatial relationships, type-based relationships are introduced to capture semantic similarities among urban patrol cases. Events belonging to the same category generally have similar risk characteristics, disposal procedures, and response requirements. Therefore, for each node, the geographically nearest nodes belonging to the same case category are selected as its semantic neighbors. The semantic adjacency matrix is defined as


$$\begin{array}{c} A_{ij}^{\text{sem}}=\left\{ \begin{array}{cc} \text{1}, & c_{i}=c_{j}\text{ and }\left(v_{j}\in N_{i}^{\text{sem}}\text{ or }v_{i}\in N_{j}^{\text{sem}}\right),\\ \text{0}, & \text{otherwise}, \end{array}\right. \end{array}$$ (7)

where $c_{i}$ and $c_{j}$ denote the event categories of nodes $v_{i}$ and $v_{j}$, respectively, and $N_{i}^{\text{sem}}$ denotes the semantic-neighbor set of node $v_{i}$.

Finally, the spatial and semantic relationships are integrated to generate the final case relationship graph:


$$\begin{array}{c} A_{ij}=I\left(A_{ij}^{\text{spa}}+A_{ij}^{\text{sem}}>\text{0}\right) \end{array}$$ (8)

where *A* denotes the final adjacency matrix and I(·) is the indicator function. If two nodes are connected through both spatial and type-based relationships, only one undirected edge is retained.

By combining distance-based and type-based edges, the constructed graph represents both geographic proximity and semantic relevance among urban patrol cases. Each node preserves its own attribute information while being connected to spatially related and semantically similar events. The resulting graph provides structured relational information for the subsequent graph attention-based autoencoder, which adaptively aggregates neighboring information to learn urgency-related latent representations.

#### Graph attention-based autoencoder pretraining.

After constructing the case-relationship graph, a graph attention-based autoencoder is pretrained to learn low-dimensional representations of urban patrol cases. The model consists of a graph attention encoder and an inner-product decoder. The encoder integrates node attributes with information from spatially and semantically related neighboring cases, whereas the decoder reconstructs the original graph structure from the learned embeddings. Historical urgency labels are not used during the pretraining process.

Let G=(V,E,X) denote the constructed case-relationship graph, where Vis the node set, Eis the edge set, and Xis the node feature matrix. The adjacency matrix is denoted by A. Before neighborhood aggregation, self-loops are added to the adjacency matrix so that each node can retain its own attribute information:


$$\begin{array}{c} \tilde{A}=A+I \end{array}$$ (9)

where I is the identity matrix.

Unlike conventional graph convolutional networks that use fixed aggregation weights, the graph attention encoder adaptively evaluates the importance of different neighboring nodes. For the l-th graph attention layer, the node representation is updated through attention-weighted neighborhood aggregation:


$$\begin{array}{c} h_{i}^{\left(l+\text{1}\right)}=\sigma \left(\sum_{j\in N_{i}}\alpha_{ij}^{\left(l\right)}W^{\left(l\right)}h_{j}^{\left(l\right)}\right) \end{array}$$ (10)

where $N_{i}$ denotes the neighborhood of node i, $\alpha_{ij}^{\left(l\right)}$ is the normalized attention coefficient between nodes i and j, $W^{\left(l\right)}$ is a learnable transformation matrix, and σ(·) represents the activation function. Through this mechanism, the encoder assigns greater weights to neighboring cases that provide more useful contextual information for representation learning.

After the stacked graph attention layers, the latent representation of node iis denoted by $z_{i}$. The latent representations of all nodes form the embedding matrix Z. Each node embedding jointly contains the original attributes of the corresponding urban patrol case and the structural information aggregated from its neighboring cases.

An inner-product decoder is employed to reconstruct the adjacency matrix from the learned embeddings:


$$\begin{array}{c} \hat{A}=\text{sigmoid}\left(ZZ^{T}\right) \end{array}$$ (11)

where $\hat{A}$ denotes the reconstructed adjacency matrix. During pretraining, the encoder and decoder parameters are optimized by minimizing the difference between the original and reconstructed adjacency matrices. Mean squared error is adopted as the graph reconstruction loss in accordance with the experimental configuration.

The graph attention-based autoencoder is pretrained using only the graph reconstruction objective. No cluster assignments, pseudo-labels, or historical urgency labels are involved in this stage. The purpose of pretraining is to preserve the attribute and structural information of the case-relationship graph and to obtain a stable latent representation for subsequent clustering optimization.

After pretraining, K-means is applied to the learned embeddings to initialize the cluster centers. The pretrained encoder parameters are retained and remain trainable during the subsequent joint optimization stage. Therefore, graph autoencoder pretraining serves as the initialization stage of the proposed deep graph clustering framework rather than as an independent feature-extraction procedure. The pretrained embeddings and encoder parameters are subsequently used for K-means center initialization and end-to-end clustering optimization.

#### K-means center initialization and joint clustering optimization.

After graph autoencoder pretraining, the learned embedding matrix Zis used to initialize the clustering module. K-means is first applied to the pretrained node embeddings to obtain the initial cluster centers and cluster assignments. The number of clusters is set to five according to the predefined urgency levels of urban patrol cases. K-means is used only for initialization rather than as an independent final clustering procedure [37,41,42].

Let $z_{i}$ denote the latent representation of node i, and let $\mu_{j}$ denote the center of cluster j [43].. The initial cluster centers are obtained by minimizing the within-cluster distance:


$$\begin{array}{c} \underset{{\left\{\mu_{j}\right\}}_{j=\text{1}}^{K}}{\text{min}}\sum_{i=\text{1}}^{N}\underset{j}{\text{min}}∥z_{i}-\mu_{j}{∥}_{\text{2}}^{\text{2}} \end{array}$$ (12)

where N is the number of nodes and K is the number of clusters. The cluster centers obtained from K-means are used as the initial values of the trainable clustering centers in the subsequent optimization stage. This initialization provides a more stable clustering structure than randomly initialized centers.

Based on the initialized centers, each node embedding is assigned to its nearest cluster center. The corresponding cluster assignment is defined as:


$$\begin{array}{c} {\tilde{y}}_{i}=\text{arg}\underset{j}{\text{min}}∥z_{i}-\mu_{j}{∥}_{\text{2}}^{\text{2}} \end{array}$$ (13)

where ${\tilde{y}}_{i}$ denotes the pseudo-label of node i. These assignments are regarded as pseudo-labels rather than ground-truth urgency labels. Historical urgency labels are not involved in pseudo-label generation or model optimization.

The pseudo-labels are used to introduce a clustering constraint into the latent representation learning process. The clustering objective minimizes the distance between each node embedding and its assigned cluster center:


$$\begin{array}{c} L_{clu}=\frac{\text{1}}{N}\sum_{i=\text{1}}^{N}∥z_{i}-\mu_{{\tilde{y}}_{i}}{∥}_{\text{2}}^{\text{2}} \end{array}$$ (14)

This objective encourages node embeddings assigned to the same cluster to become more compact in the latent space. Meanwhile, the graph reconstruction objective is retained to preserve the attribute and structural information contained in the original case-relationship graph.

The overall objective of the joint optimization stage combines the graph reconstruction loss and clustering loss:


$$\begin{array}{c} L=L_{rec}+\lambda L_{clu} \end{array}$$ (15)

where $L_{rec}$denotes the graph reconstruction loss, $L_{clu}$denotes the clustering loss, and λis a balancing coefficient controlling the contribution of the clustering objective.

During joint optimization, the graph encoder, decoder, and cluster centers are updated simultaneously. The reconstruction loss prevents the learned embeddings from deviating excessively from the original graph structure, whereas the clustering loss guides the encoder to generate more compact and separable node representations. The pseudo-labels are periodically recalculated using the updated embeddings and cluster centers. Between two pseudo-label update steps, the current assignments are kept fixed while the model parameters are optimized through backpropagation.

The optimization process alternates between pseudo-label updating and model parameter optimization. As the latent representations are progressively refined, the cluster assignments become increasingly stable. Training is terminated when the proportion of changed cluster assignments falls below a predefined convergence threshold or when the maximum number of training epochs is reached.

After convergence, the final urgency-oriented cluster of node i is determined according to the nearest optimized cluster center. Historical urgency labels are used only after clustering to establish the correspondence between cluster IDs and urgency categories and to calculate external evaluation metrics. Therefore, the proposed framework maintains an unsupervised training process while jointly optimizing graph representation learning and clustering.

### Evaluation metrics

In the proposed framework, real urgency labels are not used during representation learning or clustering optimization. They are employed only for post-hoc external evaluation to assess the consistency between the clusters discovered in the latent embedding space and the recorded urgency categories. Under the transductive clustering setting, all observed urban patrol cases participate in graph representation learning and clustering optimization. After the model converges, the model parameters, cluster centers, and cluster assignments are fixed. Since cluster identifiers do not directly correspond to the recorded urgency categories, the Hungarian algorithm is used to determine the optimal correspondence between the discovered clusters and the urgency categories. The agreement between the mapped cluster assignments and the recorded urgency labels is then evaluated using Recall, Precision, accuracy (ACC), F1-score, and normalized mutual information (NMI).

Recall measures the proportion of samples belonging to an urgency category that are correctly matched to that category after cluster-to-label mapping:


$$\begin{array}{c} \text{Recall}=\frac{TP}{TP+FN} \end{array}$$ (16)

where TP denotes the number of samples correctly matched to the corresponding urgency category, and FN denotes the number of samples belonging to that category but assigned to other categories.

Precision measures the proportion of samples assigned to an urgency category that actually belong to the corresponding recorded urgency category:


$$\begin{array}{c} \text{Precision}=\frac{TP}{TP+FP} \end{array}$$ (17)

where FP denotes the number of samples incorrectly assigned to the corresponding urgency category.

Accuracy (ACC) measures the overall agreement between the mapped cluster assignments and the recorded urgency labels:


$$\begin{array}{c} \text{ACC}=\frac{\sum_{i=\text{1}}^{N}I\left({\hat{y}}_{i}=y_{i}\right)}{N} \end{array}$$ (18)

where N is the total number of samples, $y_{i}$ is the recorded urgency label of sample i, ${\hat{y}}_{i}$ is the urgency category obtained after cluster-to-label mapping, and I(·) is the indicator function.

The F1-score is the harmonic mean of Precision and Recall and provides a balanced evaluation of the agreement between the clustering results and the recorded urgency categories:


$$\begin{array}{c} \text{F}\text{1}=\frac{\text{2}\times \text{Precision}\times \text{Recall}}{\text{Precision}+\text{Recall}} \end{array}$$ (19)

Precision, Recall, and F1-score are calculated separately for each urgency category and then averaged across all categories to obtain the macro-averaged results reported in this study. This treatment assigns equal importance to each urgency category and reduces the influence of differences in category frequency.

In addition to ACC, NMI is used to evaluate the performance of the proposed graph clustering framework. NMI is a commonly used clustering-quality metric that measures the similarity between the discovered cluster assignments and the recorded urgency categories. It is defined as


$$\begin{array}{c} \text{NMI}\left(C,Y\right)=\frac{\text{2}I\left(C;Y\right)}{H(C)+H(Y)} \end{array}$$ (20)

where C denotes the clustering result, Y denotes the recorded urgency categories, I(C;Y) represents the mutual information between C and Y, and H(C) and H(Y) are their corresponding entropies. The value of NMI ranges from 0 to 1, and a higher value indicates stronger agreement between the discovered cluster structure and the recorded urgency categories. Unlike ACC, Precision, Recall, and F1-score, NMI is calculated directly from the original cluster assignments and does not require Hungarian matching.

Because all observed nodes participate in representation learning and clustering optimization, these metrics reflect the external clustering agreement on the complete observed event graph rather than predictive performance on previously unseen cases.

## Results

### Experimental setting

The proposed framework was implemented using PyTorch and PyTorch Geometric. All experiments were conducted on a workstation equipped with an NVIDIA GPU. The model was trained using a two-stage optimization strategy, including graph attention-based autoencoder pretraining and K-means-initialized end-to-end clustering optimization.

In the first stage, the graph attention-based autoencoder was pretrained in an unsupervised manner to learn latent representations of urban patrol events. The encoder captures node attributes and graph structural information, while the decoder reconstructs the case-relationship graph. In the second stage, K-means clustering was applied to the pretrained embeddings to initialize the cluster centers. The obtained cluster assignments were then used as pseudo-labels to guide the refinement of node representations. Through joint optimization of representation learning and clustering objectives, the model progressively improves the compactness and separability of the learned clusters.

The detailed hyperparameter settings are summarized in Table 2. The encoder consists of two graph attention layers, with a hidden dimension of 64 and a latent embedding dimension of 16. RMSprop was adopted as the optimizer with an initial learning rate of 0.005 and a weight decay coefficient of 0.00005. The number of clusters was set to five according to the predefined urgency categories. The clustering loss weight, pseudo-label update interval, and convergence criterion were predefined according to preliminary unsupervised experiments and were kept fixed throughout all experiments.

**Table 2:** Network structure parameters for this method.

Parameter	Setting
Framework	Graph attention-based autoencoder with deep clustering
Graph encoder	Two-layer GAT
Hidden dimension	64
Latent embedding dimension	16
Activation function	ReLU
Decoder	Inner-product decoder
Number of clusters	5
Cluster initialization	K-means
Optimizer	RMSprop
Initial learning rate	0.005
Weight decay	0.00005
Dropout rate	0.1
Reconstruction objective	Graph reconstruction
Clustering objective	Pseudo-label guided clustering
Clustering loss weight	λ = 0.3
Maximum training epochs	200

Since the proposed method is an unsupervised clustering framework, historical urgency labels were not involved in model training or optimization. The labels were only used after clustering to establish the correspondence between the discovered clusters and urgency categories through Hungarian matching and to calculate external clustering evaluation metrics.

### Experimental analysis

To comprehensively evaluate the proposed clustering-based urgency classification framework, multiple complementary metrics are adopted, including Macro-Recall, Macro-Precision, ACC, F1, and NMI. ACC measures the overall agreement between the mapped cluster assignments and the recorded urgency categories, whereas Macro-Precision, Macro-Recall, and F1 evaluate category-level performance while reducing the influence of differences in category frequency. NMI further measures the agreement between the original cluster structure and the recorded urgency categories without requiring cluster-to-label matching. Therefore, the proposed method is evaluated from multiple perspectives rather than relying solely on clustering accuracy. The proposed method was compared with BIRCH [44], K-means [45], K-means++ [46], ISODATA [47], RMSC [48] and TADW [49] on the complete urban patrol event graph under the same transductive clustering protocol.

As shown in Table 3, the proposed method achieved the best overall performance across all five metrics, with a Macro-Recall of 0.7458, Macro-Precision of 0.7465, ACC of 0.7424, F1 of 0.7403, and NMI of 0.4517. TADW was the strongest baseline, achieving a Macro-Recall of 0.6841, Macro-Precision of 0.6986, ACC of 0.6774, F1 of 0.6713, and NMI of 0.4110. Compared with TADW, the proposed method improved Macro-Recall, Macro-Precision, ACC, F1, and NMI by 0.0617, 0.0479, 0.0650, 0.0690, and 0.0407, respectively. These results demonstrate that the proposed framework produces clusters that show stronger agreement with the recorded urgency categories than those generated by the compared methods.

**Table 3:** Performance metrics of the seven algorithms.

Method	Recall	PREC	ACC	F1	NMI
K-Means	0.2042	0.6258	0.2331	0.0851	0.0124
K-Means++	0.5146	0.5761	0.5093	0.5104	0.2656
ISODATA	0.2841	0.2911	0.2935	0.2583	0.0277
BIRCH	0.4714	0.4975	0.4868	0.4677	0.2109
RMSC	0.4874	0.5585	0.5158	0.3911	0.3141
TADW	0.6841	0.6986	0.6774	0.6713	0.4110
**Ours**	**0.7458**	**0.7465**	**0.7424**	**0.7403**	**0.4517**

Traditional clustering methods showed relatively limited performance. K-means obtained a Macro-Precision of 0.6258 but considerably lower Macro-Recall, ACC, F1, and NMI values of 0.2042, 0.2331, 0.0851, and 0.0124, respectively. The large difference between Precision and Recall indicates that K-means produced an imbalanced clustering structure and failed to adequately represent all urgency categories. K-means++ improved the initialization of the cluster centers and consequently achieved better overall performance, with an ACC of 0.5093 and a F1 of 0.5104. Nevertheless, its performance remained substantially lower than that of the proposed method.

ISODATA achieved the lowest overall performance among the adaptive clustering methods, with an ACC of 0.2935, a F1 of 0.2583, and an NMI of 0.0277. BIRCH produced more balanced results, achieving an ACC of 0.4868 and a F1 of 0.4677, but its relatively low NMI of 0.2109 indicates limited agreement between its clustering structure and the recorded urgency categories. These findings suggest that directly clustering the original event attributes is insufficient for representing the complex relationships among urban patrol cases.

RMSC achieved an ACC of 0.5158 and an NMI of 0.3141, indicating that multi-view information can improve the discovery of the overall cluster structure. However, its F1 was only 0.3911, suggesting that the correspondence between the resulting clusters and individual urgency categories remained uneven. TADW achieved the strongest performance among the baseline methods, showing that incorporating graph structural information and node-associated attributes is beneficial for urgency-oriented clustering. However, TADW separately learns graph embeddings before clustering and does not jointly refine the representations according to the clustering objective.

The superior performance of the proposed method can be attributed to the coordinated use of case-relationship modeling, graph-based representation learning, and clustering-oriented joint optimization. The case-relationship graph enables the model to integrate individual event attributes with spatial and semantic relationships among cases. The graph autoencoder pretraining stage preserves the attribute and structural information of the event graph, while K-means initialization provides a stable initial clustering structure. During joint optimization, the reconstruction objective maintains the graph information, whereas the center-based clustering objective encourages the learned embeddings to form more compact and separable clusters.

Overall, the comparative results demonstrate that conventional clustering methods have difficulty capturing the structural and contextual dependencies among urban patrol cases. By jointly modeling node attributes, case relationships, and clustering-oriented representations, the proposed method obtains more coherent urgency-related clusters and achieves better external clustering performance on the complete observed event graph.

Fig 5 shows the confusion matrices of all methods, where rows denote true urgency categories and columns denote clustered labels after Hungarian matching. A stronger diagonal indicates better clustering consistency.

**Fig 5:**
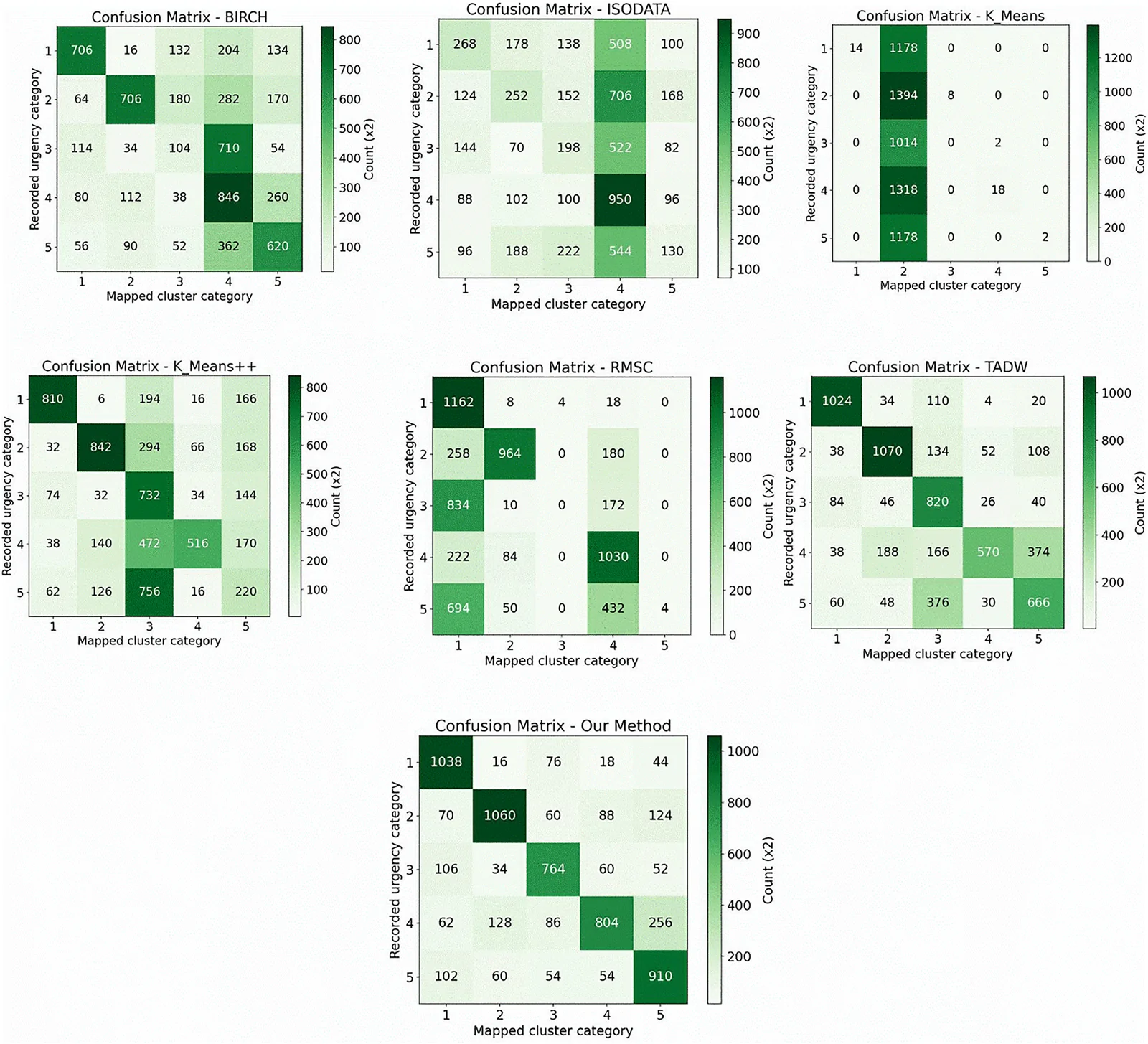
Clustering performance of the methods.

Traditional methods suffer from clear category imbalance. BIRCH and ISODATA show strong bias toward specific clusters, especially category 4, leading to large off-diagonal errors. K-means++ improves diagonal structure for low-urgency categories but confuses higher-urgency cases. RMSC performs unevenly, failing to correctly separate categories 3 and 5. TADW achieves more balanced results, with recalls of 85.91%, 76.32%, 80.71%, 42.66%, and 56.44% for categories 1–5. However, it still confuses categories 3–5, especially between adjacent high-urgency levels.

In contrast, the proposed method produces the most concentrated diagonal distribution, with recalls of 87.92%, 76.32%, 74.41%, 57.63%, and 76.61%. It significantly improves categories 4 and 5 compared with TADW, increasing recall from 42.66% to 57.63% and from 56.44% to 76.61%, respectively. Although category 3 is slightly lower than TADW, overall performance is more balanced across all classes.

A small amount of confusion remains between categories 4 and 5, likely due to their similar characteristics. Nevertheless, the proposed method still retains the highest diagonal dominance among all methods. Overall, the results are consistent with Table 5, showing that joint modeling of node attributes and case relationships effectively improves clustering quality and produces urgency structures that better match the recorded urgency categories.

### Ablation experiment

To systematically evaluate the contribution of each key component in the proposed framework, a comprehensive set of ablation experiments was designed and conducted. These experiments aim to isolate the effect of individual modules and verify their necessity in improving clustering performance for emergency urgency classification. Specifically, we considered several representative variants, including: (1) removing the graph autoencoder pretraining stage to assess its role in providing a stable initialization for representation learning; (2) removing the clustering loss to examine the impact of explicit clustering supervision during optimization; (3) constructing the graph using only spatial edges to evaluate the importance of geographic proximity information; (4) constructing the graph using only semantic edges to test the contribution of event-type similarity; (5) applying K-means directly on the learned embeddings from the GAT autoencoder without performing subsequent joint optimization, in order to verify the effectiveness of end-to-end refinement between representation learning and clustering objectives.

In all ablation settings, only the targeted component was modified, while all other experimental configurations, including network architecture, hyperparameters, training strategy, and graph construction rules, were strictly kept consistent with the full model. This controlled design ensures that any observed performance variations can be attributed solely to the removed or replaced component, thereby providing a fair and reliable comparison. The detailed quantitative results of all variants are reported in Table 4, which clearly illustrates the contribution of each module to the overall performance of the proposed method.

**Table 4:** Results of the ablation experiments.

Variant	Recall	PREC	ACC	NMI	F1
**Full model**	**0.7458**	**0.7465**	**0.7424**	**0.4517**	**0.7403**
Without graph autoencoder pretraining	0.5989	0.6671	0.6095	0.3173	0.5821
Without clustering loss	0.7226	0.7210	0.7202	0.4206	0.7162
Spatial edges only	0.7269	0.7286	0.7286	0.4195	0.7209
Semantic edges only	0.5882	0.5777	0.5847	0.3192	0.5641
GAT autoencoder + K-means without joint optimization	0.7142	0.7142	0.7117	0.4085	0.7080

The ablation results demonstrate that all major components contribute to the performance of the proposed framework. The full model achieves the best overall results, with a Recall of 0.7458, Precision of 0.7465, ACC of 0.7424, NMI of 0.4517, and F1 of 0.7403. Removing graph autoencoder pretraining causes a substantial performance degradation, reducing ACC, NMI, and F1 to 0.6095, 0.3173, and 0.5821, respectively. This indicates that pretraining is critical for learning stable and discriminative representations before clustering optimization.

The graph-relation ablations further show that spatial relationships play a more important role than semantic relationships in the current dataset. The spatial-edge-only variant achieves an ACC of 0.7286 and a F1 of 0.7209, whereas the semantic-edge-only variant obtains substantially lower values of 0.5847 and 0.5641, respectively. Nevertheless, the full model consistently outperforms the spatial-only variant, indicating that semantic relationships provide complementary information when combined with spatial proximity.

Removing the clustering loss also leads to a consistent decline in performance, while directly applying K-means to the pretrained GAT embeddings without joint optimization results in an ACC of 0.7117, an NMI of 0.4085, and a F1 of 0.7080. These results demonstrate that fixed embeddings alone are insufficient for optimal clustering. Jointly refining the encoder representations and cluster centers enables the latent space to become more compatible with the clustering objective. Overall, graph autoencoder pretraining and spatial relationship modeling provide the strongest individual contributions, while semantic relationships, the clustering loss, and joint optimization further improve clustering quality.

### Hyperparameter sensitivity analysis

To further investigate the robustness of the proposed framework and clarify the influence of the main architectural hyperparameters, sensitivity experiments were conducted on the number of GAT layers, hidden dimension, and latent embedding dimension. These parameters respectively affect the range of neighborhood information aggregation, the intermediate representation capacity of the encoder, and the dimensionality of the latent clustering space.

A one-factor-at-a-time strategy was adopted to isolate the effect of each hyperparameter. For the GAT-layer sensitivity experiment, the hidden dimension and latent embedding dimension were fixed at 64 and 16, respectively, while the number of GAT layers was varied among 1, 2, and 3. For the hidden-dimension sensitivity experiment, the number of GAT layers was fixed at 2 and the latent embedding dimension was fixed at 16, while the hidden dimension was varied among 16, 32, 64, and 128. For the latent-embedding-dimension sensitivity experiment, the number of GAT layers was fixed at 2 and the hidden dimension was fixed at 64, while the embedding dimension was varied among 8, 16, 32, and 64. In all experiments, the clustering-loss weight was fixed at λ = 0.3, and all other settings, including the graph construction strategy, optimizer, learning rate, and training procedure, were kept unchanged. Historical urgency labels were used only for post-hoc external evaluation and were not involved in model training or parameter optimization.

As shown in Table 5, the number of GAT layers has a noticeable influence on clustering performance. With a single GAT layer, the model achieved an ACC of 0.7091, an NMI of 0.4228, and a F1 of 0.7075. Increasing the network depth to two layers improved these metrics to 0.7424, 0.4517, and 0.7403, respectively, indicating that additional neighborhood aggregation enables the model to capture more informative contextual dependencies among urban patrol cases. However, further increasing the depth to three layers reduced ACC, NMI, and F1 to 0.7186, 0.4189, and 0.7185, respectively. This suggests that excessively increasing the propagation depth does not provide additional benefits and may introduce redundant neighborhood information or representation smoothing, thereby reducing the discriminability of the learned latent features. Overall, the two-layer GAT provides the best balance between neighborhood information aggregation and representation discriminability and is therefore adopted as the default encoder architecture in this study.

**Table 5:** Sensitivity analysis of the number of GAT layers.

Number of GAT layers	Recall	PREC	ACC	NMI	F1
1	0.7140	0.7276	0.7091	0.4228	0.7075
**2**	**0.7458**	**0.7465**	**0.7424**	**0.4517**	**0.7403**
3	0.7241	0.7240	0.7186	0.4189	0.7185

Table 6 shows that the hidden dimension has a noticeable influence on the clustering performance. When the hidden dimension was increased from 16 to 32, the overall performance decreased, with ACC dropping from 0.7414 to 0.7300, NMI from 0.4482 to 0.4371, and F1 from 0.7397 to 0.7272. Increasing the hidden dimension to 64 substantially improved the performance, achieving the best results across all evaluated metrics, including a Recall of 0.7458, Precision of 0.7465, ACC of 0.7424, NMI of 0.4517, and F1 of 0.7403. However, further increasing the hidden dimension to 128 did not provide additional benefits and instead caused a slight performance decline. These results suggest that an excessively large hidden space may introduce redundant information or increase optimization difficulty. Therefore, a hidden dimension of 64 was adopted as the default setting, providing an effective balance between representation capacity and clustering performance.

**Table 6:** Sensitivity analysis of the hidden dimension.

Hidden dimension	Recall	PREC	ACC	NMI	F1
16	0.7444	0.7451	0.7414	0.4482	0.7397
32	0.7331	0.7304	0.7300	0.4371	0.7272
**64**	**0.7458**	**0.7465**	**0.7424**	**0.4517**	**0.7403**
128	0.7401	0.7425	0.7402	0.4449	0.7344

Table 7 presents the sensitivity analysis of the latent embedding dimension, with the number of GAT layers fixed at 2 and the hidden dimension fixed at 64. When the embedding dimension was set to 8, the model achieved a Recall of 0.7323, Precision of 0.7329, ACC of 0.7307, NMI of 0.4337, and F1 of 0.7276. Increasing the embedding dimension to 16 substantially improved the overall clustering performance, yielding a Recall of 0.7458, Precision of 0.7465, ACC of 0.7424, NMI of 0.4517, and F1 of 0.7403. Further increasing the embedding dimension to 32 or 64 did not lead to consistent improvements across the evaluated metrics. Although the 32-dimensional setting achieved marginally higher Recall and F1 values, the 16-dimensional setting provided comparable overall performance with a more compact latent representation. Therefore, a latent embedding dimension of 16 was adopted in the final model.

**Table 7:** Sensitivity analysis of the latent embedding dimension.

Embedding dimension	Recall	PREC	ACC	NMI	F1
8	0.7323	0.7329	0.7307	0.4337	0.7276
16	**0.7458**	**0.7465**	**0.7424**	**0.4517**	**0.7403**
32	0.7463	0.7442	0.7424	0.4499	0.7409
64	0.7458	0.7465	0.7424	0.4517	0.7403

## Discussion

Recent studies have further advanced deep learning-based assessment and classification by improving backbone architectures, clustering mechanisms, and selection strategies. In terms of backbone architecture, CNN-based models such as ResNet50 and EfficientNet have been widely used because of their strong hierarchical feature extraction ability and computational efficiency. ResNet 50 introduces residual learning to ease the training of deep neural networks, while EfficientNet improves the balance between accuracy and efficiency through compound scaling of network depth, width, and resolution [50,51]. In contrast, ViT-based models divide images into patch tokens and use self-attention mechanisms to capture long-range dependencies, providing an alternative visual backbone for image classification tasks [52].

More recent architectures have also been proposed for remote sensing and hyperspectral image classification. SimPoolFormer introduces a two-stream vision transformer architecture for hyperspectral image classification, aiming to improve representation learning while reducing the computational burden of conventional transformer-based models [53]. Tri-CNN adopts a three-branch CNN structure to enhance hyperspectral image classification through multi-branch feature extraction and fusion [54]. These studies indicate that backbone architecture plays an important role in improving feature representation quality, especially for image-based classification tasks.

Recent clustering-based methods also provide useful insights for unsupervised classification and representation learning. DBSCAN is a classical density-based clustering method that can identify clusters with arbitrary shapes and handle noisy samples [55]. Building on the idea of density-based structure discovery, recent unsupervised domain adaptation research for remote sensing scene classification proposed an adaptive incremental density-based clustering strategy combined with multi-objective optimization, Pareto-front-driven feature selection, and uncertainty-aware pseudo-labeling to improve target-domain structure discovery and reduce pseudo-label noise [56]. Similarly, adaptive clustering-guided multi-scale integration has been used for traffic density estimation in remote sensing images, showing that clustering can guide region selection and multi-scale feature integration in remote sensing analysis [57].

Different from these image-oriented classificationand domain adaptation studies, the present work focuses on urban patrol case urgency classification based on structured tabular event records. The input data in this study are not remote sensing images but structured patrol case records containing event category, occurrence time, spatial coordinates, administrative-region information, and other pre-decision contextual attributes. Therefore, instead of directly adopting visual backbones such as ViT, ResNet50, or EfficientNet, this study constructs an case-relationship graph from raw patrol case attributes and uses a graph attention-based autoencoder to jointly learn node attributes and inter-case relationships. The learned embeddings are then clustered in a self-supervised manner to identify urgency-related groups. Thus, while recent studies provide useful references for backbone design, adaptive clustering, and selection strategies, our work extends clustering-based representation learning to an urban governance scenario by modeling relational dependencies among patrol cases.

The experimental results demonstrate that the proposed self-supervised deep graph clustering framework can discover urgency-related structures from urban patrol event records. The proposed method achieved a Macro-Recall of 0.7458, a Macro-Precision of 0.7465, an ACC of 0.7424, a F1 of 0.7403, and an NMI of 0.4517. TADW was the strongest baseline, with corresponding values of 0.6841, 0.6986, 0.6774, 0.6713, and 0.4110. These results indicate that the proposed method produces clusters that show stronger agreement with the recorded urgency categories than those generated by the compared methods.

The performance improvement can be attributed to the coordinated use of case-relationship graph construction, graph-based representation learning, and clustering-oriented joint optimization. Conventional clustering methods operate mainly on the original event attributes and cannot explicitly represent contextual dependencies among cases. In contrast, the proposed framework models urban patrol cases as an attributed graph and integrates event attributes with spatial and semantic relationships. The graph attention-based autoencoder preserves node-content and graph-structure information, while the center-based clustering objective encourages the learned representations to form more compact and separable groups.

The ablation results further clarify the contributions of the main components. Removing graph autoencoder pretraining caused the largest reduction in ACC, NMI, and F1, indicating that pretraining provides a stable representation for subsequent clustering optimization. The spatial-edge-only variant performed substantially better than the semantic-edge-only variant, suggesting that geographic proximity provides particularly informative contextual relationships in the current dataset. Nevertheless, combining spatial and semantic edges produced better results than using spatial edges alone, showing that event-type similarity provides complementary information. The lower performance of the model without the clustering loss and that of the GAT autoencoder followed directly by K-means also supports the contribution of clustering-oriented joint optimization.

Only attributes available before urgency classification were used as model inputs, including the reported event category, occurrence time, geographic location, administrative-region information, and other valid pre-decision contextual attributes. The recorded urgency labels and variables generated during or after urgency classification, including response priority, assigned urgency level, disposal outcome, processing status, and response-related outcomes, were excluded from graph construction, representation learning, and clustering optimization. This feature-selection strategy was adopted to reduce the risk of target leakage and ensure that the reported clustering performance was based on pre-decision event information.

Several sources of uncertainty should be considered when interpreting the results. First, the input records may contain missing attributes, inconsistent event descriptions, and inaccurate temporal or geographic information. Although preprocessing was conducted to reduce these effects, residual data-quality issues may influence feature representation and graph construction. Second, the recorded urgency labels used for post-hoc external evaluation were obtained from the historical administrative records in the original dataset and may still contain subjective judgments or operational inconsistencies. Third, the constructed graph depends on spatial proximity and event-type similarity, and different neighborhood settings may produce different graph structures and clustering results.

In addition to uncertainties related to the input data and graph construction, the proposed framework is also affected by several model and optimization parameters, including the number of spatial and semantic neighbors, network depth, hidden dimension, latent embedding dimension, clustering-loss weight, pseudo-label update interval, learning rate, and convergence threshold. In this study, targeted one-factor-at-a-time sensitivity analyses were conducted for the main architectural hyperparameters, including the number of GAT layers, hidden dimension, and latent embedding dimension. The results show that a two-layer GAT with a hidden dimension of 64 and a latent embedding dimension of 16 provides the best overall performance among the evaluated configurations. These findings indicate that the proposed framework is reasonably robust to moderate architectural changes, while inappropriate network depth or representation dimensionality may degrade clustering performance. However, an exhaustive sensitivity analysis covering all model and graph-construction parameters was not performed. In particular, the effects of the numbers of spatial and semantic neighbors, clustering-loss weight, pseudo-label update interval, learning rate, and convergence threshold were not systematically explored. Therefore, the selected configuration should be regarded as an effective setting for the current dataset rather than a universally optimal configuration. Future studies should further investigate these parameters across different cities, observation periods, and graph structures.

This study also has several limitations. The dataset contains 6,126 urban patrol cases collected from a single city over a limited observation period. Therefore, the cross-city and cross-temporal generalizability of the proposed method has not yet been empirically validated. In addition, the current framework represents temporal information through static node features and does not explicitly model the dynamic evolution of urban patrol events. Because all observed nodes participate in representation learning and clustering optimization, the reported metrics reflect external clustering agreement on the complete observed event graph rather than predictive performance on previously unseen cases. The proposed method should therefore be interpreted as an unsupervised clustering-based urgency classification framework rather than a complete replacement for supervised or operational decision-making systems.

Regarding scalability, the proposed event graph connects each case only with a predefined number of spatially or semantically related neighbors rather than constructing a fully connected graph. Consequently, when the neighborhood size is fixed, the graph remains sparse and the number of edges increases approximately linearly with the number of event nodes. The GAT encoder subsequently performs message passing only over these observed edges, which limits unnecessary computational overhead. Nevertheless, the present study does not provide systematic runtime and memory benchmarks on substantially larger urban datasets. For large-scale city-wide applications, graph construction and full-graph optimization may still impose considerable computational and memory costs. Future work will investigate neighbor sampling, graph partitioning, mini-batch training, and incremental graph updating to improve scalability to larger urban event datasets.

Future studies should evaluate the framework using larger, multi-region, and cross-temporal datasets. For large-scale urban datasets, neighbor sampling, graph partitioning, mini-batch optimization, and incremental graph updating will also be investigated to improve computational scalability. Dynamic graph neural networks may be introduced to model the temporal evolution of urban events, while adaptive graph-construction strategies may reduce the dependence on predefined neighborhood rules. Semi-supervised or active-learning strategies could also be investigated when a small number of reliable urgency annotations are available.

## Conclusions

This study proposes a self-supervised deep graph clustering framework for urban patrol case urgency classification. An attributed case-relationship graph is first constructed from raw patrol records to represent both individual case characteristics and contextual dependencies among cases. A graph attention-based autoencoder is then pretrained to learn latent node representations by integrating node attributes with graph structural information. Based on the pretrained embeddings, K-means is employed to initialize the cluster centers, after which representation learning and clustering are jointly optimized to progressively refine the latent space and cluster assignments. Historical urgency labels are not used during model training and are employed only for external evaluation. The main findings of this study can be summarized as follows:

- A graph attention–based autoencoder is designed to jointly integrate node content and graph structure into a unified latent representation, enabling effective deep feature learning on attributed graphs.
- A novel goal-oriented framework for attribute graph clustering is proposed, in which the encoder-derived embeddings directly guide the clustering process, leading to consistently improved clustering performance.
- The proposed method achieved a Recall of 0.7458, Precision of 0.7465, ACC of 0.7424, NMI of 0.4517, and F1 of 0.7403. Compared with TADW, the strongest baseline, ACC, NMI, and F1 were improved by 0.0650, 0.0407, and 0.0690, respectively.

In future work, we plan to incorporate multimodal information such as textual descriptions and on-site images to enrich node representations. Additionally, we will replace the static graph with dynamic graph neural networks to capture the temporal evolution of urban cases. Finally, we will integrate interpretability techniques together with online learning strategies to support transparent and continuously adaptive deployment in real urban management systems.
